# Potential Pitfalls in Membrane Fouling Evaluation: Merits of Data Representation as Resistance Instead of Flux Decline in Membrane Filtration

**DOI:** 10.3390/membranes11070460

**Published:** 2021-06-22

**Authors:** Bastiaan Blankert, Bart Van der Bruggen, Amy E. Childress, Noreddine Ghaffour, Johannes S. Vrouwenvelder

**Affiliations:** 1 Water Desalination and Reuse Center (WDRC), Biological and Environmental Science and Engineering Division (BESE), King Abdullah University of Science and Technology (KAUST), Thuwal 23955-6900, Saudi Arabia; bastiaan.blankert@kaust.edu.sa (B.B.); noreddine.ghaffour@kaust.edu.sa (N.G.); 2 Department of Chemical Engineering, KU Leuven, Celestijnenlaan 200F, 3001 Leuven, Belgium; bart.vanderbruggen@kuleuven.be; 3 Faculty of Engineering and the Built Environment, Tshwane University of Technology, Private Bag X680, Pretoria 0001, South Africa; 4 Astani Department of Civil and Environmental Engineering, University of Southern California, Los Angeles, CA 90089, USA; amyec@usc.edu; 5 Department of Biotechnology, Faculty of Applied Sciences, Delft University of Technology, Van der Maasweg 9, 2629 HZ Delft, The Netherlands

**Keywords:** membrane-fouling performance indicators, normalized flux decline, membrane resistance

## Abstract

The manner in which membrane-fouling experiments are conducted and how fouling performance data are represented have a strong impact on both how the data are interpreted and on the conclusions that may be drawn. We provide a couple of examples to prove that it is possible to obtain misleading conclusions from commonly used representations of fouling data. Although the illustrative example revolves around dead-end ultrafiltration, the underlying principles are applicable to a wider range of membrane processes. When choosing the experimental conditions and how to represent fouling data, there are three main factors that should be considered: (I) the foulant mass is principally related to the filtered volume; (II) the filtration flux can exacerbate fouling effects (e.g., concentration polarization and cake compression); and (III) the practice of normalization, as in dividing by an initial value, disregards the difference in driving force and divides the fouling effect by different numbers. Thus, a bias may occur that favors the experimental condition with the lower filtration flux and the less-permeable membrane. It is recommended to: (I) avoid relative fouling performance indicators, such as relative flux decline (*J/J_0_*); (II) use resistance vs. specific volume; and (III) use flux-controlled experiments for fouling performance evaluation.

## 1. Introduction

Membrane filtration plays an important role in the current and future sustainable supply of clean water. Some examples in the current state of the art are: desalination of brackish water and seawater [1,2], pretreatment for desalination [3], removal of micro-pollutants in drinking water production [4], and membrane bioreactors (MBR) [5,6]. In addition, new membrane processes are being investigated, such as forward osmosis (FO) [7], pressure-retarded osmosis (PRO) [8,9], membrane distillation (MD) [10], and hybrid processes [11]. Aside from water purification, other industries also utilize membrane to separate unwanted components from water, or to concentrate water-based product streams; for example: concentration of milk and whey, clarification of juice, and production of protein concentrates [12]. 

All these membrane processes have in common that their performance declines due to the accumulation of retained foulants on or in the membrane [12,13,14,15,16,17]. In industrial practice, the production rate is normally driven by demand. This results in flux-controlled operation, comprising intervals with constant flux and occasional adaptation of the set-point. Fouling is primarily experienced as an increase of the required driving force, resulting in higher energy consumption. In addition, fouling mitigation is an integral part of membrane-system design and operation. Membrane fouling contributes significantly to capital and operational expenses. Therefore, there is a real and clear need for prediction and control of membrane fouling [18].

Membrane fouling can be categorized into scaling, biofouling, and accumulation of rejected matter (e.g., particulate fouling, organic fouling, and colloidal fouling) [19]. The mechanism may be further distinguished based on where the fouling occurs with respect to the membrane surface and pores into external fouling, internal fouling (e.g., pore clogging), and pore blocking [20].

To evaluate the effectiveness of fouling mitigation strategies, it is desirable to effectively establish the fouling rate during filtration and the fouling state after cleaning. Establishing the fouling rate typically involves a combination of an experimental protocol and a graphical representation of the obtained data and/or a calculated fouling indicator.

In a laboratory environment, constant pressure filtration is relatively easy to perform, requiring only a gas cylinder with a pressure regulator, pressure gauge, feed vessel, membrane cell, and an electronic balance, where only the weight measurement has to be logged. Conversely, flux-controlled (e.g., constant flux) operation requires more dedicated and integrated equipment, including, for example, a controllable pump, flow-transmitter, pressure transmitter, and an automated control system. Thus, it is natural that constant-pressure fouling experiments are encountered more frequently in literature. Either the flux or the driving force may be chosen, and the other variable follows from the properties of the membrane. Thus, in these experiments, fouling is experienced as flux decline. In addition, when the driving force is constant and initially equal, differences in the filtration flux naturally occur, making it difficult to compare different experimental runs. Thus, flux decline is often normalized, by dividing the flux by its initial value, with the intention to make comparison possible. The studies that report normalized flux data are too numerous to list, and we do not intend to criticize authors or studies by citing specific examples.

Fouling indices are a combination of a standardized experimental protocol and data interpretation. The silt density index (SDI) is a commonly used fouling index. The experimental procedure and calculation of the SDI have similarities to a normalized flux-decline analysis. It has been shown that this indicator is intrinsically flawed [21,22,23,24]. Alternatively, the experimental procedure and calculation methods can be aimed to identify a fouling model parameter; for example, the modified fouling index (MFI) [25] and measurement of compressible cake parameters [26]. However, these methods rely on an assumed fouling mechanism, dead-end filtration, and specific membranes.

There are numerous ways to conduct fouling experiments, and there are multiple ways to represent the data. The combination of the experimental protocol and data representation aims to meet the following objectives:

- To provide a correct interpretation of the amount of fouling and the impact of fouling on performance;
- To characterize the fouling mechanism and to quantify fouling parameters;
- To isolate effect of manipulated operational conditions or evaluated fouling mitigation measures, such that the observation is primarily a consequence of the investigated factor.

The objective of this paper is to present and discuss a couple of examples that illustrate some of the potential pitfalls of fouling data representation and to provide recommendations for better representation of fouling data. The followed approach is to benchmark several combinations of experimental protocols (constant flux and constant pressure) and data representations. By simulating experiments, the difference in performance is a priori known from the model parameters, and it can be evaluated if this known difference is reflected in the resulting graphs. Basic fouling models for low-pressure dead-end filtration are used, because the difference in fouling effect and overall performance is unambiguous and can be understood from a small number of parameters. These model parameters were chosen with the objective to illustrate potential pitfalls.

## 2. Background

The fouling performance indicators that were considered in the example were flux (J), transmembrane pressure (TMP), resistance (R), and permeability (L), as well as their relative value (e.g., $J/J_{0}$) and their change (e.g., $R-R_{0}$). If osmotic pressure difference may be neglected, the relation between transmembrane pressure, viscosity (μ), resistance, and filtration flux is given by Darcy’s law as follows [19]:

(1) $$R=\frac{TMP}{\mu J}$$

The permeability is also frequently used as a fouling performance indicator. In the context of this paper, it is given by [19]:

(2) $$L=\frac{J}{TMP}$$

The independent variables considered were time (t) and specific volume (v). The specific volume is the cumulative filtered volume per membrane area, which can be found from integrating the filtration flux:

(3) $$\frac{dv}{dt}=J$$

The fouling state of the membrane represents the amount of fouling material that is present on the membrane surface or in the pores. When the exact composition of the feed is unknown, it is convenient to express the fouling state relative to the feed water. Thus, the fouling state is defined as the amount of feed water that contains the same amount of foulants as the fouling layer. The fouling parameters are a combination of the foulant concentration and properties. In dead-end filtration, this fouling state is equal to the filtered volume [20,27].

In this work, basic fouling models were employed so that the effect of the fouling parameters was unambiguous and could be easily understood. Therefore, complicating factors such as fouling layer compression, membrane deformation, and flux-dependent rejection of foulants were not included. The fouling models used were: ideal cake filtration, cake enhanced concentration polarization, and complete blocking.

### 2.1. Ideal Cake Filtration

In this paper, ideal dead-end cake filtration is one of the fouling models used as an example. This fouling mechanism follows the resistance in series assumption, where the total resistance is the sum of the membrane resistance ($R_{M}$) and the cake resistance (αv):

(4) $$R=R_{M}+\alpha v$$

Here, the cake resistance is proportional to the fouling state, with the specific cake resistance (*α*) as a proportionality parameter [20].

### 2.2. Cake-Enhanced Concentration Polarization

With porous membranes (MF/UF), the osmotic pressure may normally be neglected; however, in certain cases, where the feed water contains a significant concentration of macromolecules, this may not be the case. If osmotic pressure and concentration polarization in the cake may not be neglected, the transmembrane pressure has to be corrected for the osmotic pressure difference at the membrane. The effect of the fouling layer consists of hydraulic resistance and an increase of the osmotic pressure due to cake-enhanced concentration polarization [28,29]. When it is assumed that the osmotic pressure of the permeate is negligible, the filtration flux may be given by:

(5) $$J=\frac{TMP-\Pi_{F}\beta }{\mu \left(R_{M}+\alpha v\right)}$$

For simplicity, it is assumed that the concentration polarization outside of the fouling layer may be neglected. The concentration polarization factor of the fouling layer (β), which can be expressed by a structural parameter (S) and a diffusion constant (D), expressing the hindered diffusion rate in the fouling layer [30]:

(6) $$\beta =e^{\frac{JS}{D}}$$

Generally, the osmotic pressure at the membrane is not observed in experiments. If the resistance is calculated from the applied transmembrane pressure and the known feed osmotic pressure, the apparent resistance can be found by rearranging Equation (5):

(7) $$R=\frac{TMP-\Pi_{F}}{\mu J}=R_{M}+\alpha v+\Pi_{F}\frac{\beta -1}{\mu J}$$

Thus, compared to ideal cake filtration, cake-enhanced concentration polarization adds an additional term that depends exponentially on the fouling state and the filtration flux. This description of concentration polarization implies a steady state, which is not applicable for dead-end filtration. Thus, for cake-enhanced concentration polarization, an additional assumption is made that the deposition rate is equal to the dead-end deposition rate.

It is assumed that the structural parameter is proportional to the amount of fouling [28,31]. Thus, the structural parameter is the product of the specific structural parameter *s* and the specific volume. The specific cake resistance and the specific structural parameter are both a result of the thickness and structure of the fouling layer. To estimate their relative values, the specific cake resistance is described according to the Hagen–Poiseuille relation [19]:

(8) $$\alpha v=\frac{8\tau \delta }{r^{2}\epsilon }$$

where τ is the tortuosity of the pores in the fouling layer and r is their radius, while ϵ is the porosity of the fouling layer and δ is its thickness. The specific structural parameter (*s*) may be given by [32]:

(9) $$sv=\frac{\delta \tau }{\epsilon }$$

Thus, the specific cake resistance is related to the specific structural parameter via the pore radius, as follows:

(10) $$s=\frac{\alpha r^{2}}{8}$$

### 2.3. Complete Blocking 

Complete blocking was also used as an example. In this model, every particle that is retained participates in blocking pores of the membrane. Thus, the total resistance is proportional to the membrane resistance, and this mechanism does not satisfy the resistance in series assumption. The fouling parameter (*v_x_*) is a critical specific volume, namely the specific volume of feed water that contains enough particles to completely block the membrane. Thus, the resistance for a membrane fouled by the complete blocking mechanism may be given by [20]:

(11) $$R=\frac{R_{M}}{1-\frac{v}{v_{x}}}$$

## 3. Results

In this example, the aim of the simulated experiment was to compare two different membranes. There are many factors that influence which membrane is selected for a certain application, such as permeate quality, energy consumption, price, lifespan, and fouling propensity. Within this study, only the operational performance or fouling propensity was considered. Thus, the primary aim of the simulated experiments was to establish which membrane fouled more. Furthermore, the simulated experiments consisted of a single run of a limited duration. It is often beneficial to consider longer runs and multiple runs under different conditions, and to evaluate fouling reversibility. However, the scope of this study was limited to evaluation of a single filtration run.

The fouling behavior is described by ideal dead-end cake filtration, with and without concentration polarization, and complete pore blocking, where the parameters are chosen such that membrane A is superior to membrane B in terms of both membrane resistance and fouling propensity. A positive correlation between membrane resistance and fouling rate was found by comparing the fouling rates of different membranes [33]. Furthermore, that study found that the magnitude of the correlation differed between feed-water sources. One possible explanation is that the pore size affected the amount of retained material that accumulated and the morphology of the fouling layer.

### 3.1. Constant-Pressure Experiment with Equal Pressure (Cake Filtration)

**Figure 1:**
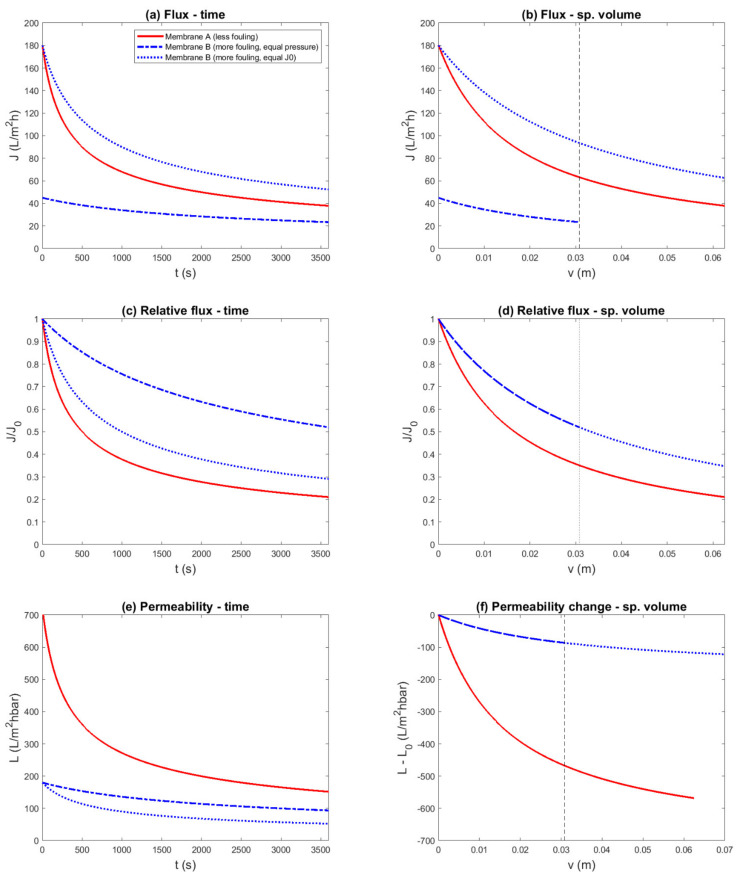
Simulated constant-pressure experiment, aiming to compare two membranes with equal transmembrane pressure (0.25 bar) and equal initial flux (TMP_B_ = 1 bar), and several flux- and permeability-based data representations. The example was constructed such that membrane A was superior to membrane B, due to both a lower membrane resistance ($R_{MA}=0.5\times 10^{12}$ m^−1^ vs. $R_{MB}=2.0\times 10^{12}$ m^−1^) and a lower fouling rate ($\alpha_{A}=3.0\times 10^{13}$ m^−2^ vs. $\alpha_{B}=6.0\times 10^{13}$ m^−2^). Different representations (**a**—Flux–Time; **b**—Flux–Specific Volume; **c**—Relative Flux–Time; **d**—Relative Flux–Specific Volume; **e**—Permeability–Time; **f**—Premeability Change–Specific Volume) were comp—red to evaluate if the known difference in fouling propensity was reflected: (**c**,**d**,**f**) suggest a conclusion contradicting the underlying parameters.

**Figure 2:**
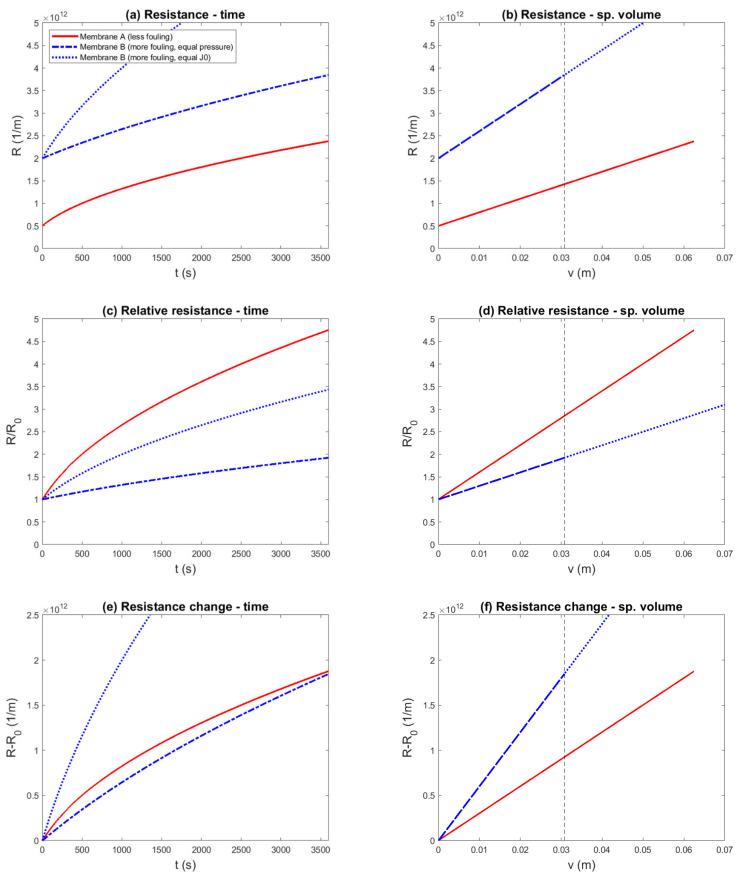
Simulated constant-pressure experiment, aiming to compare two membranes with equal transmembrane pressure (0.25 bar) and equal initial flux (TMP_B_ = 1 bar), and several pressure- and resistance-based data representations. The example was constructed such that membrane A was superior to membrane B, due to both a lower membrane resistance ($R_{MA}=0.5\times 10^{12}$ m^−1^ vs. $R_{MB}=2.0\times 10^{12}$ m^−1^) and a lower fouling rate ($\alpha_{A}=3.0\times 10^{13}$ m^−2^ vs. $\alpha_{B}=6.0\times 10^{13}$ m^−2^). Different representations (**a**—Resistance–Time; **b**—Resistance –Specific Volume; **c**—Relative Resistance–Time; **d**—Relative Resistance–Specific Volume; **e**—Resistance Change–Time; **f**—Resistance Change–Specific Volume) were compared to evaluate if the known difference in fouling propensity was correctly reflected: (**c**–**e**) suggest a conclusion contradicting the underlying parameters.

Ideal cake filtration was chosen as a fouling model, in which membrane A was superior compared to membrane B in terms of both membrane resistance ($R_{MA}=0.5\times 10^{12}$ m^−1^ vs. $R_{MB}=2.0\times 10^{12}$ m^−1^) and fouling propensity ($\alpha_{A}=3.0\times 10^{13}$ m^−2^ vs. $\alpha_{B}=6.0\times 10^{13}$ m^−2^). These values could be encountered with UF of surface water. A constant-pressure experiment was simulated, and the results are shown in 12 different representations: Figure 1 shows the flux plots, and Figure 2 shows the resistance plots.

Two membranes may be compared by evaluation of flux decline with the same constant driving force. However, it is well known that two membranes should not be compared if they operate at a vastly different flux [34]. Thus, an experimental protocol in which the initial flux is set equal for both membranes is often favored. This implies that a different transmembrane pressure is chosen for each membrane. Here, we considered both ways of conducting a constant-pressure experiment.

Flux as a function of time, as it was measured during the experiment, is shown in Figure 1a. By comparing the flux decline with equal driving force, this figure shows that the overall performance of membrane A was superior to the performance of membrane B, due to the higher filtration flux during the entire run. It was not obvious how to assess the difference in fouling rate: visually, the flux decline of membrane A appeared to be more severe; however, it could also be argued that the two lines cannot be compared in this way. Conversely, if both membranes were compared based on equal initial flux, it appeared that the flux decline curves could be compared, and that membrane B fouled less compared to membrane A. Thus, this experimental protocol with equal initial flux and flux-decline representation led to the conclusion that contradicted the underlying facts. The reason was that due to the higher transmembrane pressure for membrane B, a similar amount of fouling resulted in a higher filtration flux.

When the initial flux is different, it is common to plot the normalized or relative flux instead. The relative flux as a function of time is plotted in Figure 1c. The normalization forced the initial points of the curves to be identical. The consequence, as was intended, was that it now appears that all the lines may be compared. However, in this representation, the observed fouling rate of membrane B seemed to be roughly half of the fouling rate of membrane A with the same TMP, which contradicted the facts underlying this example. This demonstrated that a membrane that is inferior in terms of both membrane permeability and fouling propensity can appear superior by relative flux-decline representation.

Due to the different values of the filtration flux, it was clear that at the same point of time, the filtered volume differed between the different variants of the experiment. To take this into account, fouling performance indicators discussed above were also plotted as a function of the specific volume (Figure 1b,d). In these representations, it is apparent that in the same time, the filtered volume for membrane B with equal TMP was smaller. Thus, the assessment was made by comparing different parts of the fouling curves, compared to the representations with time on the *x*-axis, which was relatively more favorable for the more permeable membrane A and the higher TMP run with membrane B. Nevertheless, the flux-decline plot was still difficult to interpret, and the relative flux-decline plots still gave the misleading impression that the inferior membrane B performed better.

Permeability is another fouling performance indicator that can be considered when the TMP or the initial flux differ (Figure 1e). This representation is similar to the flux decline curve. By normalizing the data with respect of the driving force, much of the difference between the curves of membrane B was eliminated. Similarly to the flux-decline curve, it was difficult to assess the difference in fouling rate between membrane A and B. One could consider plotting the relative permeability (*L/L_0_*), which would reduce to the relative flux. Alternatively, the permeability loss as a function of specific volume is plotted in Figure 1f. Taking the difference of the permeability (as in actual value minus the initial value) also forced the lines to start at the same point, again giving the impression that the lines were comparable. In this representation, the permeability loss of membrane A appeared much more severe than membrane B, which contradicted the facts.

It is interesting to note that while in Figure 1c,e, it appears that the fouling rate was different for both experiments with membrane B, it is clear in Figure 1d,f that this was only related to volume. Thus, in the case of ideal cake filtration, there was no difference between normalizing the flux in the data representation versus equalizing the initial flux by adapting the TMP. The remaining difference was attributed to the difference in filtered volume.

Resistance as a function of time is plotted in Figure 2a. This representation shows that the overall performance of membrane A was superior, due to the lower resistance during the entire run. However, when comparing membranes A and B based on equal TMP, the fouling behavior was difficult to assess; visually, the fouling rate appeared similar. This was made more clear by plotting the change in resistance as a function of time (Figure 2e). Indeed, the fouling rates appeared similar, which again contradicted the facts underlying this example. However, when the two membranes were compared based on equal initial flux, the difference in fouling rate started to become clear.

One might also consider making it easier to compare filtration curves by normalizing the resistance with respect to its initial value. The relative resistance is plotted in Figure 2c. This chart misleadingly suggests that fouling in membrane A was much more severe than in membrane B.

The resistance is plotted as a function of specific volume in Figure 2b. In this representation, the membrane resistance can be identified as the y-intercept, and the fouling rate can be identified as the slope. To emphasize the difference in the fouling rate, the change in resistance is shown in Figure 2f. This representation makes the difference in slope more clearly visible and shows that membrane A fouled less than membrane B, in accordance with the fouling parameters.

In summary, 12 representations of the same simulated constant-pressure experiment were shown, to evaluate if the known difference in fouling parameters was reflected in the data representation. Four of these; namely, the flux vs. time (Figure 1a), flux vs. specific volume (Figure 1b), permeability vs. time (Figure 1e), and resistance vs. time (Figure 2a) plots, correctly showed that the overall performance of membrane A was superior to membrane B. However, in these plots, the fouling propensity could not be clearly assessed. The resistance vs. volume plot (Figure 2b) was able to show both overall performance and the fouling propensity. The resistance change vs. volume plot (Figure 2f) made the difference in fouling propensity more clear, but gave no information about the overall performance. The other seven plots gave the misleading impression that membrane B was superior to membrane A.

### 3.2. Implication for Comparing Different Processes

It can be interesting to compare the fouling propensity of different membrane processes treating the same feed water. Within the context of the current example, one might want to assess the fouling rate of a hypothetical novel membrane process, involving, for example, a pressure, concentration, and/or temperature difference, and compare this to UF. It was assumed that, due to the complexity of the novel system, it was only experimentally feasible to maintain a constant driving force and observe the filtration flux. As there was no clear way to define a ‘resistance’ for such a process, only flux decline was evaluated. In the selection of experimental conditions, the initial filtration flux of the reference ultrafiltration system, operating at constant pressure was chosen to be equal to the initial flux of the novel membrane process.

**Figure 3:**
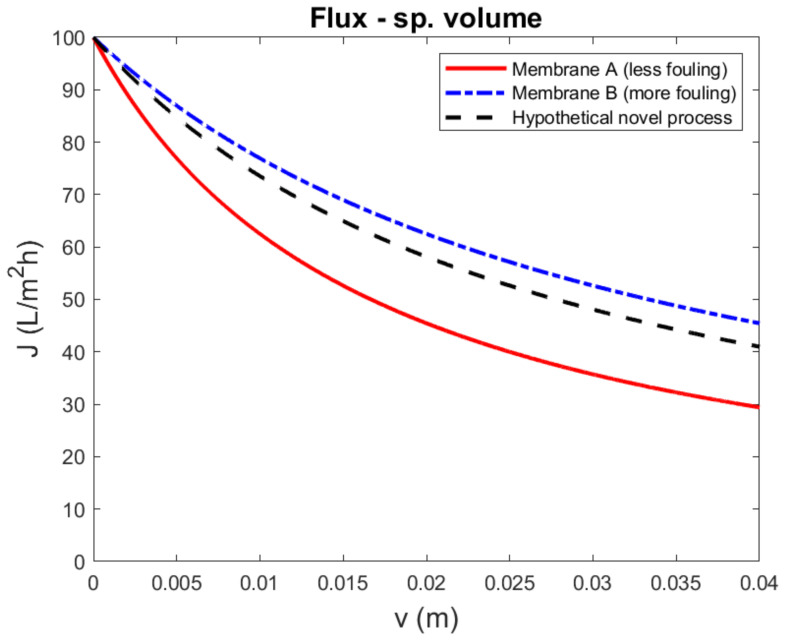
Simulated flux-decline curves to compare a hypothetical novel membrane process to ultrafiltration. The example was constructed such that membrane A was superior to membrane B, due to both a lower membrane resistance ($R_{MA}=0.5\times 10^{12}$ m^−1^ vs. $R_{MB}=2.0\times 10^{12}$ m^−1^) and a lower fouling rate ($\alpha_{A}=3.0\times 10^{13}$ m^−2^ vs. $\alpha_{B}=6.0\times 10^{13}$ m^−2^). The transmembrane pressure was chosen to have identical initial flux (TMP_A_ = 0.14 bar, TMP_B_ = 0.56 bar). The fouling mechanism of the novel membrane was assumed to be unknown, but constructed to lie between the curves of membrane A and B. If the novel membrane process was compared with a more-fouling and less-permeable reference membrane, it appeared that the fouling rate of the novel process was worse; conversely, if the less-fouling, more-permeable reference membrane was used, it appeared that the novel process fouled less.

The previous section compared two similar membranes and demonstrated that if two membranes were compared based on a constant-pressure experiment with equal initial flux, the membrane with the higher membrane resistance had a slower flux decline. To illustrate the possible implication for comparing different processes, it was assumed that the flux-decline curve of the novel membrane process, with unknown fouling mechanism, happened to be somewhere between the curves of membrane A and B, as shown in Figure 3. Thus, if the novel membrane process was compared to ultrafiltration, using the less-permeable and more-fouling membrane B as reference, it appeared that ultrafiltration was superior in terms of fouling rate, and vice versa. More generally, this implied that, whatever the flux decline curve is of a novel process, it is in theory possible to find a reference membrane that has a sufficiently low membrane resistance to compare unfavorably; and conversely, it is possible to find a reference membrane that has a sufficiently high membrane resistance to compare favorably. The first will require a higher transmembrane pressure compared to the latter. However, the difference in driving force was not reflected in the evaluation, and it was not obvious how the driving force of the novel process compared to the transmembrane pressure.

A similar reasoning can be applied to the novel membrane process, without understanding precisely how its driving force should be interpreted. If the process is relatively inefficient, it requires a relatively large driving force to attain the desired initial filtration flux. Thus, if a certain amount of the available driving force is needed to overcome fouling, the remaining driving force for transport through the membrane is still large and relatively unaffected. Conversely, if the novel process is highly efficient, the same amount of fouling, reducing driving force with the same amount, will affect the remaining driving force significantly. Thus, flux-decline experiments, with different driving forces, have a bias toward the less-efficient membrane or system.

### 3.3. Constant-Flux Experiment (Cake Filtration)

**Figure 4:**
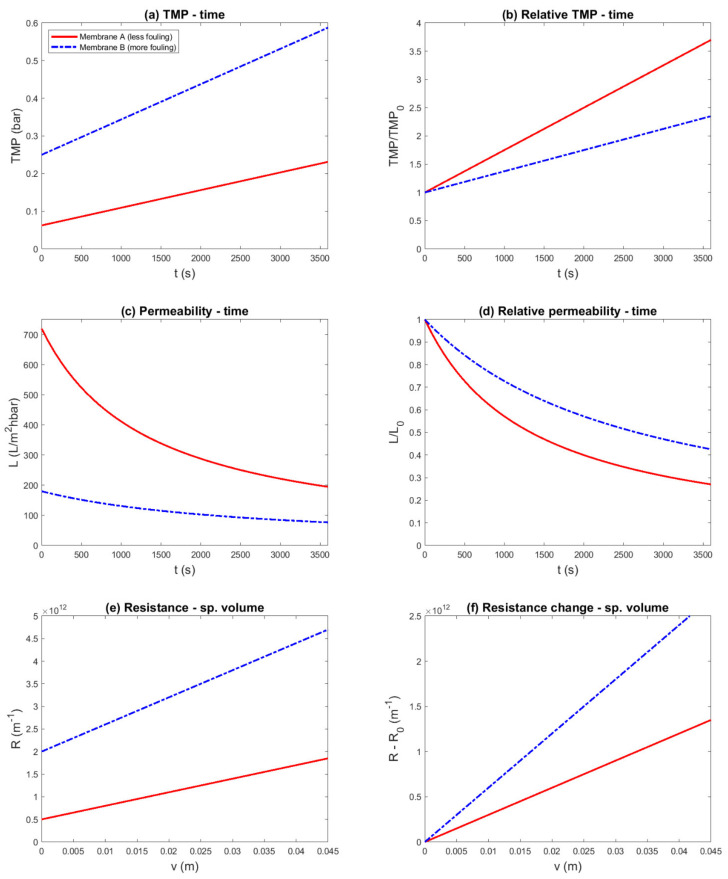
Simulated constant-flux experiment, aiming to compare two membranes. The example was constructed such that membrane A was superior to membrane B, due to both a lower membrane resistance ($R_{MA}=0.5\times 10^{12}$ m^−1^ vs. $R_{MB}=2.0\times 10^{12}$ m^−1^) and a lower fouling rate ($\alpha_{A}=3.0\times 10^{13}$ m^−2^ vs. $\alpha_{B}=6.0\times 10^{13}$ m^−2^). Different representations (**a**—Transmembrane Pressure (TMP)–Time; **b**—Relative TMP–Time; **c**—Permeability–Time; **d**—Relative Permeability–Time; **e**—Resistance–Specific Volume; **f**—Resistance Change–Specific Volume) were compared to evaluate if the known difference in fouling propensity was correctly reflected: (**b**,**d**) suggest a conclusion contradicting the underlying parameters.

An alternative way to equalize the flux is to utilize a flux-controlled experiment, in which both membranes are operated at the same constant filtration flux. To maintain the constant flux, the transmembrane pressure has to be increased as the membrane fouls, and so the transmembrane pressure is the measured variable. The previous example was continued, with the same membranes and fouling rates. The results of the simulated constant-flux experiments are shown in six different representations in Figure 4a–f.

The transmembrane pressure as it would be measured during the experiment is plotted in Figure 4a. This representation allowed the difference in membrane resistance (y-intercept) and fouling rate (slope) to be identified after some calculations. Thus, in a constant-flux experiment, the measured variable is directly related to the fouling parameters.

The relative transmembrane pressure is plotted in Figure 4b. The intent of this normalization was to make the difference in fouling rate more explicit. However, in this way, the fouling rate of membrane B appeared to be lower than the fouling rate for membrane A, which again contradicted the facts underlying this example.

The permeability is plotted in Figure 4c. This figure shows that the overall performance of membrane A was superior, due to the higher permeability during the entire run. The difference in fouling behavior was not obvious: it appeared that permeability decline was more severe for membrane A, but it can also be argued that the two lines cannot be compared. Permeability was normalized to facilitate comparison; this is shown in Figure 4d. In this representation, the fouling rate of membrane A appeared worse, which contradicted the facts. Note that the permeability (‘specific flux’) decline was similar to the flux decline (Figure 2a,b), although the rate of decline was lower in the constant-flux experiment.

The resistance (Figure 4e) and resistance change (Figure 4f) were plotted against the specific volume. Note that these plots are in principle identical to Figure 2e,f. In constant-flux experiments, the specific volume is linearly related to time. Thus, in this case, there was essentially no difference between plotting against time or volume. The resistance and resistance-change plots also corresponded to the known difference in performance between the two membranes.

### 3.4. Cake-Enhanced Concentration Polarization

**Figure 5:**
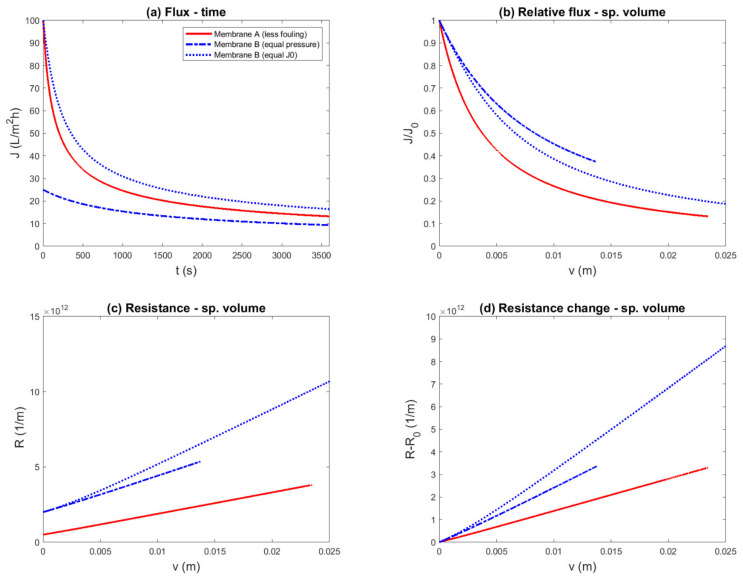
Simulated constant-pressure experiment, aiming to compare two membranes with equal transmembrane pressure (0.39 bar) or equal initial flux (TMP_A_ = 0.39 bar, TMP_B_ = 0.81 bar). The example was constructed such that membrane A was superior to membrane B, due to a lower membrane resistance ($R_{MA}=0.5\times 10^{12}$ m^−1^ vs. $R_{MB}=2.0\times 10^{12}$ m^−1^), a lower fouling rate ($\alpha_{A}=3.0\times 10^{13}$ m^−2^ vs. $\alpha_{B}=6.0\times 10^{13}$ m^−2^), and a lower structural parameter ($s_{A}=3.75\times 10^{-4}\;\mathrm{vs}.\;s_{B}=7.5\times 10^{-4}$ (r=10 nm ) and $D=1\times 10^{-10}m^{2}/s$, $\Pi_{F}=0.25\;\mathrm{bar}$ ). Different representations (**a**—Flux–Time; **b**—Relative Flux–Specific Volume; **c**—Resistance–Specific Volume; **d**—Resistance Change–Specific volume) were compared to evaluate if the known difference in fouling propensity was correctly reflected. Flux decline with equal initial flux (**a**) and relative flux decline (**b**) curves suggest a conclusion contradicting the underlying parameters.

**Figure 6:**
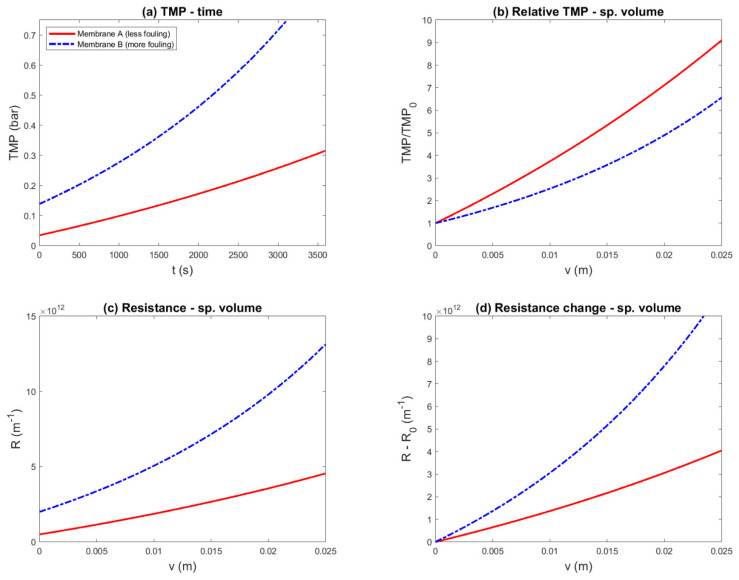
Simulated constant-flux experiment, aiming to compare two membranes. The example was constructed such that membrane A was superior to membrane B, due to a lower membrane resistance ($R_{MA}=0.5\times 10^{12}$ m^−1^ vs. $R_{MB}=2.0\times 10^{12}$ m^−1^), a lower fouling rate ($\alpha_{A}=3.0\times 10^{13}$ m^−2^ vs. $\alpha_{B}=6.0\times 10^{13}$ m^−2^), and a lower structural parameter ($s_{A}=3.75\times 10^{-4}\;\mathrm{vs}.\;s_{B}=7.5\times 10^{-4}$ (r=10 nm ) and $D=1\times 10^{-10}m^{2}/s$, $\Pi_{F}=0.25\;\mathrm{bar}$ ). Different representations (**a**—TMP–Time; **b**—Relative TMP–Specific Volume; **c**—Resistance–Specific Volume; **d**—Resistance Change–Specific volume) were compared to evaluate if the known difference in fouling propensity was correctly reflected: Relative transmembrane pressure (**b**) suggests a conclusion that contradicts the underlying parameters.

In this section, we considered that the osmotic pressure of the feed cannot be neglected, and that the concentration polarization in the fouling layer has to be taken into account. In the context of low-pressure membrane filtration, this might occur when the feed contains dissolved macromolecules, such as proteins. The results of a simulated constant-pressure experiment are shown in Figure 5, and the results of a simulated constant-flux experiment are shown in Figure 6.

The constant-pressure experimental protocol can be designed to compare the two membranes with equal transmembrane pressure (lower flux for membrane B) or with equal initial flux (higher TMP for membrane B). The flux as it would be measured is shown in Figure 5a, and the relative flux as function of specific volume is shown in Figure 5b. The plot of the relative flux decline suggests that membrane B fouled less than membrane A, contradicting the facts underlying the example.

It is further interesting to note that the relative flux-decline curves of membrane B, with equal driving force and equal initial flux, were similar. Thus, a flux-decline curve can be biased even if the initial flux is chosen equal and the filtration flux is similar during the rest of the run. Nevertheless, the experimental approach with equal initial flux was able to reduce much of the difference in filtration flux, which exponentially exacerbated the concentration polarization.

The resistance and the change in resistance are plotted in Figure 6c,d. In these plots, it is visible that membrane B fouled more than membrane A. Due to the concentration polarization, the resistance depended exponentially on the filtration flux, and the apparent fouling rate was significantly lower for the run with the lower initial flux. Thus, the comparison based on equal initial flux, with similar flux during the entire run, better reflected the difference in fouling propensity.

Alternatively, a constant-flux experiment can be used, with the benefit of ensuring that the effect of flux is constant during the experiment and equal for both membranes. The transmembrane pressure as it would be measured during such an experiment is shown in Figure 6a. It can be seen that the pressure increased exponentially due to the concentration polarization factor. Furthermore, the differences in membrane resistance and fouling rate can be recognized from the y-intercept and the difference in the slopes. However, if the transmembrane pressure was normalized (Figure 6b), the difference in fouling rate became unclear: during the experiment, it appeared that membrane A fouled slightly more, but it was also apparent that eventually the curves would cross.

The resistance and change in resistance are shown in Figure 6c,d. In the resistance chart, the difference in membrane resistance (y-intercept) and the fouling rate (slope) can be recognized. In the resistance change vs. specific volume plot of the constant flux experiment, the difference in fouling rates between the membranes could be well assessed.

### 3.5. Constant-Pressure Experiment (Complete Blocking)

In this section, complete blocking was considered as the fouling mechanism. This model assumes that all particles that are rejected completely block pores. By this assumption, the effect of fouling is multiplicative with the membrane resistance, rather than additive, as was the case for cake filtration. Thus, the resistance-in-series approach does not apply to this fouling mechanism. This property of the fouling relation suggests that it may be more appropriate to use relative fouling performance indicators. In this example, membranes A and B were used again. In complete pore blocking, the fouling parameter is defined as a critical specific volume: this represents the volume of feed water that contains enough particles to block all the pores [27], which was chosen to be twice as much for membrane A compared to membrane B. The values of the parameters were chosen so that the filtered volume was similar compared to the cake-filtration example ($v_{xA}=0.075$ m and $v_{xB}=0.0375$ m). Again, the example was created such that membrane A was superior in both aspects.

**Figure 7:**
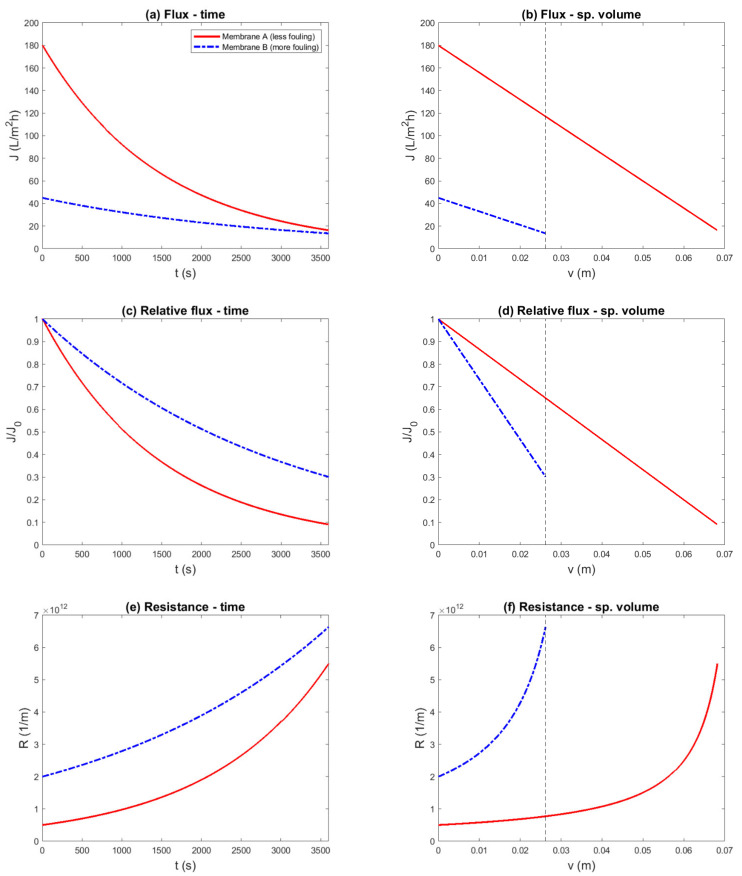
Comparison between two membranes for a single constant-pressure (0.25 bar) run with different representations of the data. The example was constructed such that membrane A was superior to membrane B, due to both a lower membrane resistance ($R_{MA}=0.5\times 10^{12}$ m^−1^ vs. $R_{MB}=2.0\times 10^{12}$ m^−1^) and a lower fouling rate ($v_{xA}=0.075$ m vs. $v_{xB}=0.0375$ m). Different representations (**a**—Flux–Time; **b**—Flux–Specific Volume; **c**—Relative Flux–Time; **d**—Relative PerFlux–Specific Volume; **e**—Resistance–Time; **f**—Resistance Change–Specific Volume) were compared to evaluate if the known difference in fouling propensity was correctly reflected: Relative flux vs. time (**c**) and resistance vs. time (**e**) suggest a conclusion contradicting the underlying parameters.

The plots of flux as a function time (Figure 7a) and the resistance as a function of time (Figure 7e) show that the overall performance of membrane A was superior to membrane B. The fouling rate was difficult to assess; however, flux decline seemed more severe for membrane A, and the resistance increase appeared similar.

The relative flux as a function of time is plotted in Figure 7c. This figure suggests that the fouling rate for membrane A was larger than the fouling rate for membrane B. Thus, this fouling data representation was not able to reflect the difference in fouling parameters, regardless of the fact that the fouling relation was proportional to the membrane resistance.

The flux (Figure 7b) and relative flux (Figure 7d) were plotted against the specific volume. In both of these graphs, the fouling parameter was equal to the x-intercept of the fouling curve. Thus, their numerical values were easily obtained by extrapolating the lines. The difference in fouling rate is not visually obvious in the flux plot. The relative flux plot shows this more clearly. However, the difference in overall performance is not visible in the latter.

Resistance was plotted as a function of specific volume in Figure 7f. This figure correctly shows both the difference in overall performance and the difference in fouling rate. The fouling parameters can be recognized as the asymptotes of the fouling curves. However, their numerical values of 0.0375 m and 0.075 m cannot be obtained easily from the graph. 

It is interesting to note that in the resistance plot (Figure 7f), the fouling curve appeared to be escalating, and around a specific volume of 0.025 m^3^/m^2^, the fouling of membrane B appeared severe, while the fouling for membrane A appeared to be minor. In contrast, in the flux representations (Figure 7b,d), the escalating character and the difference between the two membranes appeared to be much less pronounced.

**Figure 8:**
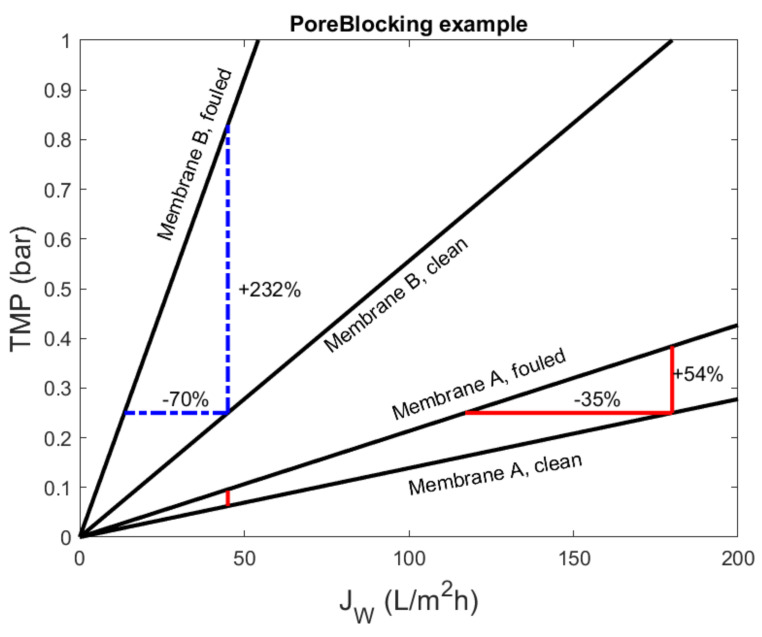
Relation between filtration flux and transmembrane pressure for clean and fouled membranes (final fouling state: *v* = 0.0262, $R_{MA}=0.5\times 10^{12}$ m^−1^, $R_{MB}=2.0\times 10^{12}$ m^−1^, $v_{xA}=0.075$ m, and $v_{xB}=0.0375$ m). In this figure, a constant-pressure experiment corresponds to moving over a horizontal line from the clean membrane to the fouled membrane. Similarly, a constant-flux experiment corresponds to a horizontal line. For every value of the filtration flux, the required transmembrane pressure (energy), for the fouled membrane A was 54% higher than for the clean membrane A (232% for membrane B).

In industrial practice, the primary objective of a membrane system is to meet a certain demand for treated water. Thus, these systems are typically flux-controlled, and the desired flux is adapted to the current demand. The resulting operation does not necessarily correspond to either constant flux or constant pressure. Rather, the fouling state of the membrane defines the relation between flux and the driving force. These relations were plotted for both membranes, clean and fouled, in Figure 8. In this plot, a constant-pressure experiment corresponded to moving over a horizontal line from the clean membrane to the fouled membrane, and a constant-flux experiment corresponded to moving upward over a vertical line. 

Permeability and flux decline are inversely related to economic performance, such as energy consumption or required membrane area. This inverse relation may make it unintuitive to relate relative flux to operational performance. For example, in Figure 7 and Figure 8, the flux decline for membrane B (70%) was twice the decline for membrane A (35%). However, for any desired filtration flux, the fouled membrane B required 232% additional pressure (energy) compared to the clean membrane (vs. 54% for membrane A). For economic performance, the amount of energy is much more important than its relative change. If both membranes operated at the same filtration flux (e.g., 45 L/m^2^h), then the difference between a clean and fouled membrane corresponded to 0.033 bar (A) and 0.58 bar (B). In other words, the additional energy corresponding to 70% flux decline for membrane B was 18× as large as the additional energy, corresponding to a 35% flux decline for membrane A.

Although the relative flux vs. specific volume plot was appropriate to determine the fouling model parameter for pore blocking, this representation does not show the severity of the fouling. The operational impact was more obvious in the resistance plots, albeit this type of plot made it more difficult to obtain the fouling parameter.

## 4. Discussion

The suitability of experimental protocols and fouling data representations, and the results of examples shown, can be explained using the following arguments:

1. Volume bias: The constituents that cause membrane fouling are contained in the feed water. Thus, the amount of fouling material on the membrane surface principally depends on the filtered volume. Equivalently, the accumulation rate (as amount of material per unit time) principally depends on the filtration flux. In experiments with constant and equal driving force, differences in flux can occur, which result in corresponding differences in the foulant mass. By plotting the results as a function of time, the difference in fouling load is neglected, and the representation is biased toward the run with the lower filtration flux (Figure 2e vs. Figure 2f).
2. Flux bias: The adverse effect of fouling is often exacerbated by a higher filtration flux, due to, for example, polarization phenomena and fouling-layer compression. Thus, even if the experiment and data representation take the difference in filtered volume into account, a bias still exists that favors the run with the lower filtration flux. Note that in our examples for ideal cake filtration and pore blocking, this did not play a role; however, in more complex fouling mechanisms, for example with cake-enhanced concentration polarization, this is highly relevant. This secondary flux effect is related to the difference in flux between two runs, and can be isolated in flux-controlled fouling experiments, in which different factors are compared on an equal filtration flux basis.
3. Normalization bias: Relative fouling performance indicators involve normalization of the data by an initial value. In essence, this corresponds to dividing the fouling resistance by the membrane resistance. Thus, this introduces a bias toward a membrane with a higher resistance, simply by dividing the effect of fouling by a larger number (Figure 1c,d). This effect also plays a role in constant-driving-force experiments, in which the initial flux is chosen to be equal and the required difference in driving force is neglected.

The arguments listed above and their relation to experimental conditions are summarized in Table 1.

In relative flux vs. time representation of a constant-driving force experiment, all three of these effects are compounded. As demonstrated in the example, the results may be distorted to an extent where conclusions can be drawn that contradict the facts. Thus, this representation should be avoided. If the initial flux and membrane resistance of the experiments are similar, the effect of normalization can be relatively small; however, in that case, the flux declines can also be compared directly. On the other hand, if the difference in initial flux is large, it seems necessary to normalize the data, but the distortion of the results (due to normalization bias) is then also significant. Relative flux decline is discussed specifically, as this representation is common. However, similar arguments hold for the less commonly encountered fouling performance indicators, such as: relative transmembrane pressure ($TMP/TMP_{0}$), relative permeability ($L/L_{0}$), permeability difference ($L-L_{0}$), permeability decline rate (dL/dt), and relative resistance ($R/R_{0}$).

The example and the arguments presented above suggest that resistance vs. specific volume is the most appropriate representation. As was the case in our examples, when the driving force is only hydraulic pressure and the fouling mechanism is related to accumulation of material, this is certainly the case. However, there are some nuances to consider.

- Fouling mechanisms that involve chemical (scaling) or biological conversion may be kinetically limited depending on the conditions. In such cases, it may be more appropriate to use time on the *x*-axis. In these cases, the concentration at the membrane surface is highly relevant, thus concentration polarization has to be taken into account carefully. In this case, it is especially recommended to utilize flux-controlled experiments, as this removes the distinction between time and specific volume, and equalizes the concentration polarization between runs and during the run.
- In some processes, there is an interplay of driving forces. For example, in membrane distillation, the transport through the membrane is driven by a vapor pressure difference, whereas the transport through the fouling layer is driven by hydraulic pressure. In addition to the hydraulic resistance of the fouling layer, fouling may also enhance polarization phenomena. In such cases, it may not be clear how the resistance of the fouling layer can be determined, or how the fouling effect can be isolated from the intrinsic membrane properties. The experimental protocols discussed in this paper relied on comparing constant-flux or constant-pressure runs. When the fouling mechanism is more complex, a single filtration run under constant conditions may not contain sufficient information about the fouling characteristics, and an extended experimental protocol may be needed [26,29].
- In crossflow filtration, the accumulation of fouling is more loosely related to the filtered volume compared to dead-end filtration. In most cases, it is expected that in a crossflow system, the fouling rate is reduced, since shear and back-diffusion can remove foulants from the fouling layer [35]. However, it is also possible that enhanced mass transfer accelerates transport of foulants toward the membrane [36]. In either case, the effect of the hydrodynamic conditions on the fouling rate is visible by evaluating the amount of fouling per filtered volume.

It should be noted that we chose the parameters deliberately to illustrate potential pitfalls in the selection of experimental procedure and fouling data representation that introduce a bias in the results. It is certainly possible that under different conditions, the introduced bias would be small enough that the interpretation of the experiment would not be affected in a meaningful way. However, in the context of a real experiment, it may be difficult to determine whether the introduced bias is small enough or not. Thus, it would be preferable to avoid introducing a bias altogether. The given examples were limited to simplified models of dead-end filtration with parameters typical for UF of surface water. Thus, the results presented cannot be directly applied to other, more complicated systems. On the other hand, if a certain approach can be shown to be biased in a simplified system, one should carefully consider if following a similar approach is suitable for a more complicated system.

## 5. Conclusions

Commonly used data representations can distort the membrane-fouling results to an extent that conclusions appear that contradict facts. Thus, the way fouling data is represented should be carefully considered.

The following recommendations are made:

- Relative (normalized) fouling performance indicators, such as relative flux ($J/J_{0}$), relative transmembrane pressure ($TMP/TMP_{0}$), relative resistance ($R/R_{0}$), and relative permeability ($L/L_{0}$) are not appropriate for fouling quantification.
- It is always appropriate to plot a measured variable (e.g., flux or pressure) as a function of time. However, that does not necessarily mean that performance can be assessed from the resulting plot. Meaningful differences between experimental runs (e.g., different pressures in flux-decline experiments) should be clearly indicated.
- Under the resistances-in-series assumption, the change in resistance ($R-R_{0}$) can be used to give a more explicit impression of the fouling by removing the contribution of the membrane. For other fouling indicators, representations based on their change should be avoided (e.g., $L-L_{0}$ and dL/dt).
- If hydraulic pressure is the main driving force and the fouling mechanism is accumulation of foulant matter from the feed water, it is recommended to represent the fouling data as resistance vs. specific volume. These conditions are commonly encountered in cases in which currently normalized flux decline is shown.
- In flux-decline experiments, differences in filtered volume and secondary effects of the difference in filtration flux should be carefully considered. It is preferable to compare experiments with different driving forces and equal initial flux, rather than experiments with equal driving forces and different initial flux. The flux-decline curve of the latter experiment has similar properties to the relative flux decline, and should not be used directly to assess fouling rate.
- In constant-flux filtration experiments, the effect of the filtration flux on fouling is controlled. Thus, the investigated effects can be better isolated. It is highly recommended that for comparison of membrane-fouling performance, tests should be performed at the same controlled flux and be evaluated for a rise in resistance and driving force.

## Figures and Tables

**Table 1:** Summary of the discussed causes for biases in different experiments. In the example given in this paper, A refers to the more-permeable membrane, and B refers to the less-permeable membrane. More generally, A can refer to the system or situation that more efficiently relates driving force to flux.

Experimental Condition	Constant Driving Force		Constant Flux
	Equal Initial Flux	Equal Driving Force	Equal Flux
Observation	B has a higher average flux	A has a higher flux	A has a lower driving force
(1) Volume bias	Favors A	Favors B	None
(2) Flux bias	Favors A	Favors B	None
(3) Normalization bias	Favors B Occurs in J(t), J(v), and relative fouling indicators	Favors B Occurs in relative fouling indicators	

## Data Availability

Data sharing not applicable.
